# Acknowledgment to Reviewers of *Journal of Intelligence* in 2021

**DOI:** 10.3390/jintelligence10010009

**Published:** 2022-01-28

**Authors:** Journal of Intelligence Editorial Office

**Affiliations:** MDPI AG, St. Alban-Anlage 66, 4052 Basel, Switzerland

Rigorous peer-reviews are the basis of high-quality academic publishing. Thanks to the great efforts of our reviewers, *Journal of Intelligence* was able to maintain its standards for the high quality of its published papers. Thanks to the contribution of our reviewers, in 2021, the median time to first decision was 38 days. The editors would like to extend their gratitude and recognition to the following reviewers for their precious time and dedication, regardless of whether the papers they reviewed were finally published:
Adam B. LockwoodJustin BrienzaAdam ChuderskiKamila UrbanAlexander ChristensenKarl SchweizerAlissa BeathKees Jan KanAndre BeauducelKimberly WingertAndré KretzschmarKristina MeyerAndrea Trubanova WieckowskiKristof KovacsAndreas DemetriouLarry M. StarrAndrew ConwayLudivine MartinAntonella LopezMaría José Gutiérrez CoboAthanasios KoutrasMarianne NolteBoris ForthmannMark LeikinBranton ShearerMarta BistronBruno DauvierMartha MichalkiewiczCarmen FerrandizMatthias ZieglerCasey L. BrownMercedes FerrandoCatrinel Haught TrompMichael LewisConnie S. BarberMichèle N. SchubigerDana JosephMiguel-Ángel Esteban-NavarroDaniel SimonetMihaela Flavia AvramDavid Aly RedvaldsenMoshe ZeidnerDavid C. GearyNicholas F. BensonDavid CropleyOliver WilhelmDiane HalpernPaola AielloElizabeth AustinPaul SilviaEmanuel JaukPhillip L. AckermanErnesto San MartinQin ZhaoEstefanía MartínRachel WilsonEsther KeulersRex JungEunice Gaerlan-PriceRobert SternbergEvgenia GkintoniRogier A. KievitFlorian SchmitzRory MacLeanFrancisco Villegas LirolaRoza LeikinGeorge SpanoudisRui Abrunhosa GonçalvesGizem HülürSara HartGordon B. SchmidtSarah DavisIan DearySelcuk AcarIsaac Nana AkuffoSerena MastriaJan-Philipp FreudensteinSergio AgnoliJeffrey M. GredleinSergio MorraJens F. BeckmannSilvia Postigo-ZegarraJens LangeSimonetta D’AmicoJillian LauerStefano A. CerriJohn MayerStephen SkalickyJonas LangSteven UmbrelloJonathan PluckerThierry LecerfJonathan WaiTobias DebatinJose M. MestreToni SchmaderJuan WangTorberg FalchJudith A. HallUlrich SchroedersJudith GlückVivian CiaramitaroJulian Schulze



